# Monocyte count and soluble markers of monocyte activation in people living with HIV and uninfected controls

**DOI:** 10.1186/s12879-022-07450-y

**Published:** 2022-05-11

**Authors:** Andreas D. Knudsen, Randa Bouazzi, Shoaib Afzal, Marco Gelpi, Thomas Benfield, Julie Høgh, Magda Teresa Thomsen, Marius Trøseid, Børge G. Nordestgaard, Susanne D. Nielsen

**Affiliations:** 1 Viro-Immunology Research Unit, Department of Infectious Diseases 8632, Rigshospitalet, University of Copenhagen, Blegdamsvej 9B, DK-2100 Copenhagen Ø, Denmark; 2 Department of Cardiology, The Heart Center, Rigshospitalet, University of Copenhagen, Copenhagen, Denmark; 3 The Copenhagen General Population Study, Department of Clinical Biochemistry, Herlev and Gentofte Hospital, Copenhagen University Hospital, Herlev, Denmark; 4 Department of Infectious Diseases, Hvidovre Hospital, University of Copenhagen, Hvidovre, Denmark; 5 Section of Clinical Immunology and Infectious Disease, Oslo University Hospital, Oslo, Norway

**Keywords:** Monocytes, HIV, Monocyte activation markers, Soluble CD14, Soluble CD163, Monocytopenia, Chronic inflammation

## Abstract

**Background:**

Monocytes play an important role in inflammation, and monocytosis and monocyte activation are features of chronic inflammation. We aimed to investigate if HIV status was associated with monocyte count and monocyte activation and to assess the relationship between monocyte count and monocyte activation markers and HIV-related factors.

**Methods:**

Persons living with HIV (PLWH) with measured monocyte count and sCD14 and sCD163 were included from the Copenhagen Comorbidity in HIV infection (COCOMO) study and matched 1:5 on sex and age with uninfected controls. In addition, 74 uninfected individuals from COCOMO with measured sCD14 and sCD163 were included. Identical protocols and equipment were used to determine monocyte counts and monocyte activation in PLWH and uninfected controls. Linear regression adjusted for age, sex, smoking and waist-to-hip-ratio was used to analyze the association between possible risk factors and monocyte outcomes.

**Results:**

We included 871 PLWH and 4355 uninfected controls. PLWH had − 0.021 [− 0.031 − 0.011] × 10^9^/L) lower monocyte count than uninfected controls, and in adjusted analyses HIV status was independently associated with − 0.035 [− 0.045, − 0.025] × 10^9^/L lower monocyte count. In contrast, PLWH had higher sCD163 and sCD14 concentrations than uninfected controls. After adjustment, HIV-status was associated with higher sCD14 and sCD163 concentrations (588 [325, 851] ng/ml, and 194 [57, 330] ng/ml, respectively).

**Conclusion:**

PLWH had lower monocyte counts than controls, but the absolute difference was small, and any clinical impact is likely limited. In contrast, concentrations of monocyte activation markers, previously implicated as drivers of non-AIDS comorbidity, were higher in PLWH than in controls.

**Supplementary Information:**

The online version contains supplementary material available at 10.1186/s12879-022-07450-y.

## Background

Persons living with HIV (PLWH) have shorter life expectancies and fewer comorbidity-free years than the uninfected population [1, 2] and a high prevalence of non-AIDS comorbidities such as metabolic and cardiovascular diseases has been reported [3–5]. This may be due to traditional risk factors such as smoking that are prevalent in PLWH [6, 7]. However, HIV-specific risk factors including immune activation and inflammation play an important role in the pathogenesis as well [8–11].

Monocytes are part of the innate immune system and an integral part of the initiation and maintenance of the acute inflammatory response. However, monocytes are also key constituents in chronic inflammation and may be a driver in the pathogenesis of inflammation-related diseases such as atherosclerosis[9, 12]. Monocytes express CD4 and may, consequently, become infected with HIV although the clinical significance of this among treated PLWH is not well-explored.

The soluble forms of monocyte surface proteins CD14 and CD163 (sCD14 and sCD163, respectively) are shed by activated monocytes and function as markers of monocyte activation and inflammation [13–16]. Studies have shown sCD14 and sCD163 to be associated with non-AIDS comorbidities in PLWH [10, 16–20], and PLWH may have higher concentrations of monocyte activation markers than age-matched uninfected controls [10, 11, 21–23]. Whether PLWH, per se*,* have higher monocyte counts and whether elevated monocyte counts contribute to the higher concentrations of monocyte activation is not known.

The purpose of this study was to determine if HIV status is independently associated with higher monocyte counts and concentration of monocyte activation markers. Furthermore, we aimed to identify both HIV-specific and HIV-unspecific risk factors associated with higher monocyte counts and concentrations of monocyte activation markers. Because of the previously reported association between HIV and higher monocyte activation [10, 11, 21–23], we hypothesized, that HIV status would be independently associated with higher monocyte count as well as with higher concentrations of monocyte activation markers.

## Methods

### Design and study population

PLWH were recruited from the Copenhagen Comorbidity in HIV infection study (COCOMO), an observational, longitudinal study designed to determine the burden of co-morbidities in PLWH [24]. Between March 2015 and November 2016, the COCOMO study included 1099 PLWH aged 20–100 years, all living in the greater Copenhagen area. Of all PLWH living in Copenhagen > 40% were included in the COCOMO study. For this study, only COCOMO participants with available monocyte count and monocyte activation markers sCD14 and sCD163 were included.

For analyses of monocyte counts, uninfected controls were recruited from the Copenhagen General Population Study (CGPS). CGPS is an observational longitudinal study, including > 110,000 participants residing in the greater Copenhagen area [25]. Participants were matched 1:5 on sex and 5-year age strata with uninfected controls from CPGS with measured monocyte count in the same period. The matching ratio was limited to 1:5 as we estimated little extra gain in statistical power with more controls (Additional file 1:Fig. S1).

COCOMO and CGPS use identical questionnaires and study protocols, but participants in CGPS have not had concentrations of monocyte activation markers measured. Thus, we additionally recruited seventy-four HIV-uninfected participants into COCOMO and measured the concentration of sCD14 and sCD163 to serve as uninfected controls for inflammatory markers only.

All participants provided written informed consent. Both the COCOMO study (H-8-2014-0004) and the CGPS study (H-KF-01-144/01) have obtained approval from the Ethics Committee of the Capital Region and from the Danish Data Protection Agency. Data are available for review at our institution upon reasonable request.

### Data sampling

Data collection was identical in COCOMO and CGPS. Data collection was identical in COCOMO and CGPS. Information on smoking and self-reported origin was obtained from questionnaires.. Height, weight, waist and hips circumference, systolic and diastolic blood pressure were measured by health professionals[24]. BMI was defined as a person’s weight in kilograms divided by the square of the person’s height in meters (kg/m^2^) according to WHO definition[26]. The waist-hip ratio (WHR) was calculated as waist circumference divided by hip circumference according to the WHO definition [27]. According to Joint National Committee guidelines, hypertension was defined as current antihypertensive treatment and/or systolic blood pressure at least 140 mmHg and/or diastolic blood pressure at least 90 mmHg [28]. Diabetes was defined as self-reported diabetes and/or antidiabetic treatment and/or plasma glucose ≥ 11.1 mmol/L [4].

Information on HIV-associated variables, including CD4 + count, CD8 + count, viral load, nadir CD4 + count and cART regimens, were retrieved from patients’ records. Low CD4 + nadir was defined as nadir CD4 + count < 200 cells/μL. For monocyte activation markers, plasma was collected and stored at − 80 °C.

### Biochemistry

Monocyte count, high sensitivity C-reactive protein (hsCRP), low-density lipoprotein (LDL) and high-density lipoprotein (HDL) cholesterol, triglycerides, total cholesterol and plasma glucose, were analyzed at a single laboratory at Herlev and Gentofte Hospital, Copenhagen University Hospital.

Monocytosis and monocytopenia were defined according to local laboratory reference as a monocyte count greater than 800/µL (> 0.8 × 10^9^/L) and lower than 200/µL (< 0.2 × 10^9^/L), respectively.

Plasma concentrations of sCD163 and sCD14 were measured using ELISA (R&D Systems, Minneapolis, Minnesota, US), using 384-plates and the combination of a SELMA pipetting robot (Jena, Germany) and a BioTek dispenser/washer (EL406, Winooski, Vermont, US). Optical density was read at 450 nm with wavelength correction set to 540 nm using an ELISA plate reader (Synergy H1 Hybrid, Biotek, Vinooski, Vermont, US).

### Statistical analyses

Continuous variables were reported as medians with interquartile ranges (IQR) and categorical variables as frequencies and percentages. To assess differences in continuous variables between PLWH and uninfected controls, Mann–Whitney *U* and *t*-tests were used as appropriate, and *χ*^2^ tests were used to assess differences in categorical variables. To analyze the association between non-HIV-related risk factors and HIV-related risk factors and monocyte count or monocyte activation markers, we used multiple linear regression adjusted for a prespecified model based on prior assumptions of likely confounders. The model included age, sex, WHR and smoking status. In a sensitivity analysis, we included origin into our prespecified model. Potential risk factors were adjusted for the model individually and one at a time. To assess the association between HIV and monocytosis or monocytopenia, we used univariable and multivariable logistic regression adjusted for the same model.

Unadjusted and adjusted numeric estimates and 95% confidence intervals, were reported for each continuous outcome variable, monocyte count and sCD163 and sCD14.

All statistical analyses were performed using R [29].

## Results

In total, 871 PLWH and 4,355 uninfected controls were included in analyses of monocyte counts. In addition, PLWH were compared with 74 uninfected controls from the COCOMO study who were included as controls in the analyses of monocyte activation markers. Characteristics of the participants are shown in Table 1 and data on missing variables are listed in Additional file 1: Table S1.

**Table 1:** Demographic and clinical information of participants

	PLWH N = 871	Uninfected controls N = 4355
Age in years*,* mean (SD)	51 (11)	52 (11)
Female sex, n (%)	121 (14%)	643 (15%)
European origin, n (%)	635 (74%)	3,992 (93%)
Non-European origin, n (%)	223 (26%)	306 (7%)
BMI kg/m^2^, mean (SD)	25 (4)	27 (4)
Underweight, n (%)	23 (2.7%)	16 (0.4%)
Normal, n (%),	466 (54%)	1645 (38%)
Overweight, n (%)	295 (34%)	1917 (44%)
Obese, n (%)	83 (10%)	766 (18%)
WHR, mean (SD)	0.9 (0.1)	0.9 (0.1)
Abdominal obese, n (%)	532 (63%)	2549 (59%)
Former smoker, n (%)	307 (35%)	1582 (36%)
Current smoker, n (%)	246 (28%)	553 (13%)
Hypertension, n (%)	364 (45%)	1586 (37%)
Diabetes, n (%)	38 (4.5%)	189 (4.4%)
Triglycerides, mM mean (SD)	2.1 (1.5)	1.9 (1.3)
HDL, mM, mean (SD)	1.2 (0.5)	1.3 (0.5)
hsCRP, mg/L, mean (SD)	2.7 (6)	2 (5)
Antilipidemics, n (%)	108 (12%)	421 (10%)
Antihypertensives, n (%)	138 (16%)	610 (14%)
Duration of cART, yr, median (IQR)	11 (5, 7)	NA
Undetectable, n (%)	831 (95)	NA
CD4 + nadir < 200cells/µL, n (%)	346 (39)	NA

### Monocyte counts in PLWH and uninfected controls

**Fig. 1:**
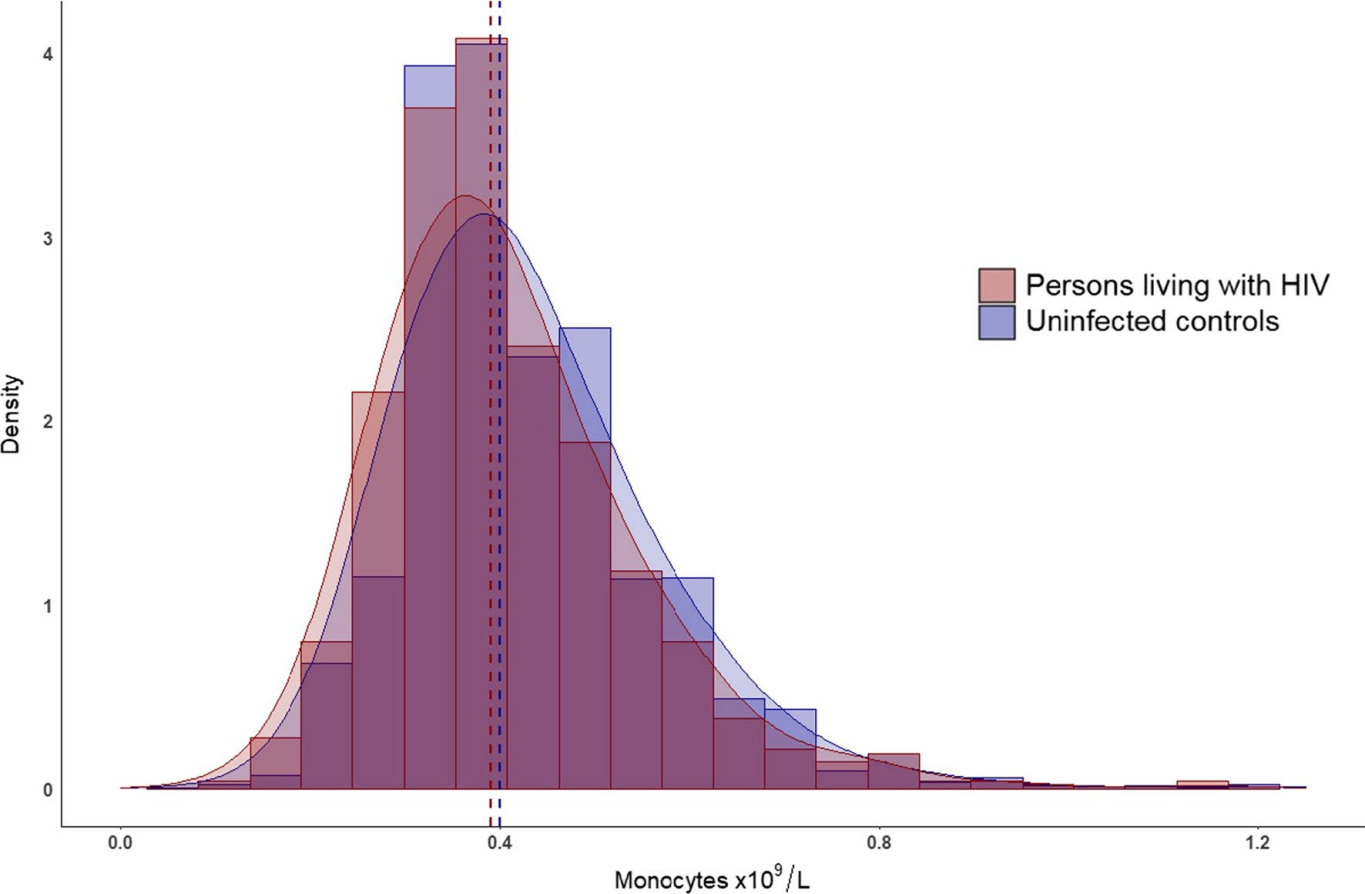
Histogram with kernel density plot of monocyte concentration in persons living with HIV and uninfected controls. The concentration of monocytes (in × 109/L) in persons living with HIV (red) and uninfected controls (blue). Dashed lines represent median concentration for persons living with HIV (red line) and uninfected controls (blue line). Although the mean monocyte count was significantly lower among persons living with HIV, the absolute difference was small

PLWH had a lower mean monocyte count than uninfected controls (0.407 × 10^9^/L (0.133) vs 0.428 × 10^9^/L (0.138), respectively, p < 0.001) (Fig. 1).

Monocytopenia was present in 15 (1.7%) of PLWH and 24 (0.6%) of uninfected controls (crude odds ratio, OR: 3.16 [1.65, 6.05] p < 0.001). Monocytosis was present in 14 (1.6%) of PLWH and in 57 (1.3%) of uninfected controls (crude OR: 1.23 [0.68, 2.22], p = 0.488).

In unadjusted analyses, female sex, non-European origin, and HDL were associated with lower mean monocyte counts and with monocytopenia. Age, WHR, current smoking, former smoking, hypertension, diabetes, hsCRP*,* and triglycerides were associated with higher mean monocyte counts (Table 2). See s Additional file 1: Table S2 for factors associated with monocytosis.

**Table 2:** Association between risk factors and monocyte count

	Unadjusted β (× 10^9^/L	Adjusted β (× 10^9^/L
HIV, yes vs. no	− 0.021 [− 0.031, − 0.011], p < 0.001	− 0.035 [− 0.045, − 0.025], p < 0.001
Age per decade	0.015 [0.011, 0.018], p < 0.001	0.008 [0.004, 0.011], p < 0.001
Female sex, yes vs. no	− 0.044 [− 0.054, − 0.033], p < 0.001	− 0.018 [− 0.029, − 0.007], p = 0.002
Origin outside of Europe, yes vs. no	− 0.015 [− 0.027, − 0.002], p = 0.019	− 0.014 [− 0.026, − 0.002], p = 0.027
WHR, per standard deviation	0.019 [0.01, 0.028], p < 0.001	0.010 [− 0.000, 0.020], p = 057
Former smoker vs. never smoker	0.025 [0.017, 0.033], p < 0.001	0.015 [0.007, 0.023], p < 0.001
Current smoker vs. never smoker	0.077 [0.066, 0.087], p < 0.001	0.069 [0.058, 0.079], p < 0.001
Hypertension, yes vs. no	0.041 [0.033, 0.049], p < 0.001	0.023 [0.015, 0.032], p < 0.001
Antihypertensives, yes vs. no	0.051 [0.041, 0.062], p < 0.001	0.032 [0.021, 0.044], p < 0.001
Diabetes, yes vs. no	0.040 [0.022, 0.059], p < 0.001	0.008 [− 0.011, 0.026], p = 0.419
HDL-cholesterol, per mM	− 0.038 [− 0.046, − 0.030], p < 0.001	− 0.022 [− 0.030, − 0.013], p < 0.001
LDL-cholesterol, per mM	0.000 [− 0.004, 0.004], p = 0.881	− 0.003 [− 0.007, 0.001], p = 0.116
Triglycerides, per mM	0.012 [0.009, 0.015], p < 0.001	0.004 [0.001, 0.007], p = 0.004
Total Cholesterol pr mM	0.001 [− 0.003, 0.004], p = 0.758	− 0.003 [− 0.006, 0.000], p = 0.068
Antilipidemic, yes vs. no	0.047 [0.034, 0.059], p < 0.001	0.022 [0.009, 0.034], p < 0.001
CD8 + cell count per 100 cells	0.007 [0.005, 0.009], p < 0.001	0.006 [0.004, 0.008], p < 0.001
CD4 + cell count per 100 cells	0.011 [0.008, 0.014], p < 0.001	0.011 [0.008, 0.014], p < 0.001
CD4 + nadir < 200cells/µL, yes vs no	0.008 [− 0.011, 0.026], p = 0.413	− 0.001 [− 0.020, 0.018], p = 0.906
HIV-RNA > 50copies/mL, yes vs no	0.019 [− 0.023, 0.061], p = 0.381	0.018 [− 0.025, 0.060], p = 0.414

Associations between independent variables and monocyte count. Left column shows unadjusted β and right column shows adjusted β

In analyses adjusted for age, sex, WHR and smoking status, HIV was associated with lower mean monocyte count (Table 2). Female sex, HDL-cholesterol and non-European origin were also associated with lower monocyte counts, although origin was not associated with monocyte counts when adjusted for HIV status. Older age, hypertension, current smoking, former smoking, hsCRP, WHR, and triglycerides were associated with higher mean monocyte counts. HIV was associated with an adjusted OR (aOR) of 3.64 [1.84, 7.21] of monocytopenia, p < 0.001, but not with monocytosis (p = 0.954). Current smoking, hsCRP, CD4 count and CD8 count were associated with higher odds of having monocytosis but no other variables were associated with either monocytopenia or with monocytosis (Table 3 and Additional file 1: Table S2, respectively).

**Table 3:** Association between risk factors and monocytopenia

	Crude odds ratio	Adjusted odds ratio
HIV, yes vs. no	3.16 [1.65;6.05], p < 0.001	3.64 [1.84, 7.21], p < 0.001
Age per decade	0.78 [0.59, 1.05], p = 0.103	0.75 [0.54, 1.04], p = 0.085
Female sex, yes vs. no	2.31 [1.15, 4.67], p = 0.019	2.21 [0.99, 4.97], p = 0.054
WHR, per standard deviation	0.12 [0.00, 5.43], p = 0.278	1.81 [0.02, 165.59], p = 0.797
Former smoker vs. never smoker	0.58 [0.28, 1.18], p = 0.132	0.53 [0.25, 1.12], p = 0.096
Current smoker vs. never smoker	0.37 [0.11, 1.24], p = 0.107	0.38 [0.11, 1.26], p = 0.114
Hypertension, yes vs. no	0.48 [0.23, 1.01], p = 0.053	0.52 [0.22, 1.21], p = 0.127
Antihypertensives, yes vs. no	0.47 [0.15, 1.54], p = 0.216	0.66 [0.19, 2.28], p = 0.508
Diabetes, yes vs. no	1.18 [0.28, 4.92], p = 0.822	1.61 [0.36, 7.17], p = 0.531
HDL-cholesterol, per mM	1.76 [0.96, 3.24], p = 0.070	1.74 [0.84, 3.62], p = 0.137
LDL-cholesterol, per mM	0.88 [0.63, 1.23], p = 0.459	0.95 [0.67, 1.35], p = 0.771
Triglycerides, per mM	0.78 [0.57, 1.08], p = 0.141	0.86 [0.62, 1.18], p = 0.345
Total Cholesterol per mM	0.93 [0.70, 1.25], p = 0.631	1.00 [0.74, 1.36], p = 0.985
Antilipidemic, yes vs no	0.72 [0.22, 2.34], p = 0.582	1.12 [0.32, 3.91], p = 0.864
High-sensitivity CRP per mg/L	0.99 [0.91, 1.08], p = 0.792	1.00 [0.93, 1.08], p = 0.991
CD8 + cell count per 100 cells	0.81 [0.67, 0.97], p = 0.026	0.86 [0.71, 1.03], p = 0.093
CD4 + cell count per 100 cells	0.88 [0.72, 1.09], p = 0.244	0.90 [0.73, 1.12], p = 0.359
CD4 + nadir < 200cells/µL, yes vs no	1.46 [0.51, 4.21], p = 0.480	1.56 [0.48, 5.11], p = 0.464
HIV-RNA > 50copies/mL, yes vs no	Model did not converge	Model did not converge

Associations between independent variables and monocytopenia. Left column shows crude (unadjusted) odds ratios and right column shows adjusted odds ratios

Within PLWH, 100 cell-increase in CD4 + and CD8 + cell counts were associated with higher monocyte counts, after adjusting for age, sex, WHR and smoking status. cART, low CD4 + nadir and previous AIDS were not associated with monocyte count, monocytopenia or monocytosis.

### Monocyte activation in PLWH and uninfected controls

**Fig. 2:**
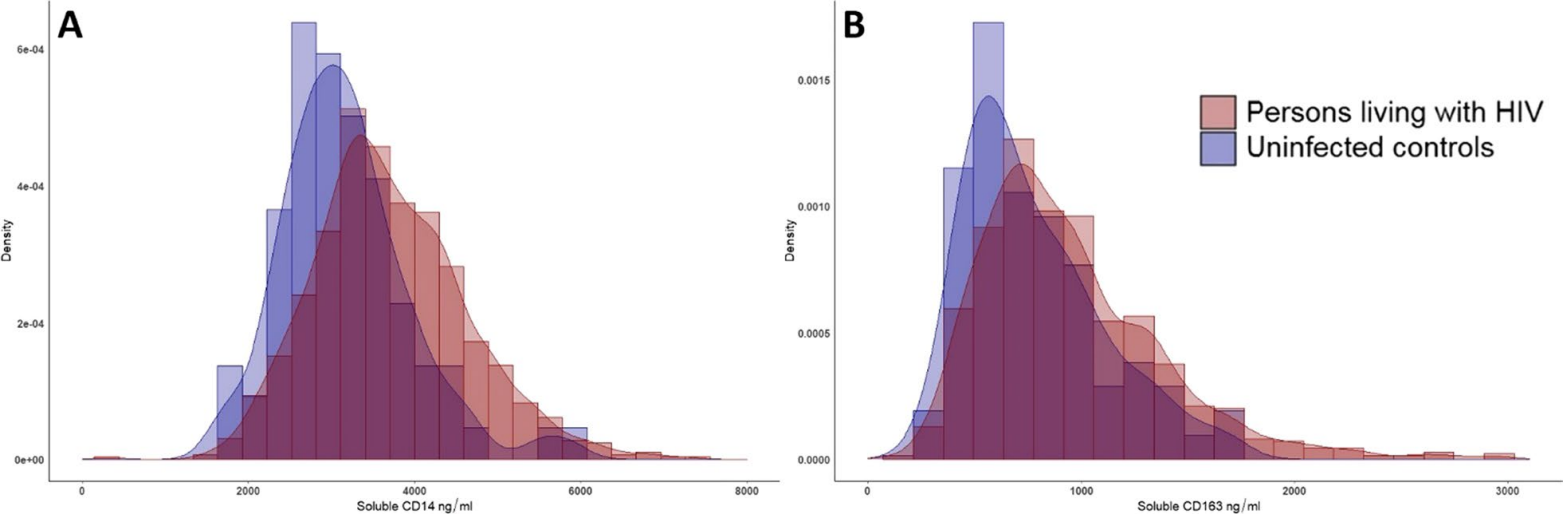
Histograms with density plots of concentrations of soluble CD163 and soluble CD14 among persons living with HIV. Density plots showing concentrations of **A** soluble CD14 and **B** soluble CD163 among persons living with HIV.”

PWLH had higher concentrations of sCD14 and sCD163 than controls (3720 (954) vs 3170 (772) ng/ml, p < 0.001, and 969 (500) vs 774 (318) ng/ml, p < 0.001; mean difference: 551 [362, 739] ng/ml, and 196 [116, 276] ng/ml, respectively) (Fig. 2). In adjusted analyses, HIV status was independently associated with (194 [57, 330] ng/ml higher sCD163 and 588 [325, 851] ng/ml higher sCD14 (p = 0.006 and p < 0.001, respectively).

### Factors associated with soluble CD163 in PLWH

In unadjusted analyses in PLWH only, age, WHR, diabetes, hsCRP, monocyte count, detectable viral load, and CD8 + cell count, were associated with higher sCD163 concentrations, while HDL, was associated with lower sCD163 concentrations.

In adjusted analyses (Table 4), female sex, WHR, diabetes, detectable viral load, CD8 + cell count and monocyte count were associated with higher sCD163 concentrations. Hypertension, HDL, and total cholesterol, were associated with lower sCD163 concentrations*.* No significant association was found between smoking status, LDL, triglyceride current CD4 + , or low CD4 + nadir and sCD163 concentrations.

**Table 4:** Association between risk factors and markers of monocyte activation

	Soluble CD163 β in ng/ml	Soluble CD14 β in ng/ml
HIV, yes vs no	194 [57, 330], p = 0.006	588 [325, 851], p < 0.001
Age per decade	− 4 [− 38, 29], p = 0.807	74 [8,139], p = 0.028
Female sex, yes vs no	125 [26, 224], p = 0.0137	237 [41, 432], p = 0.018
WHR, per standard deviation	100 [63, 138], p < 0.001	48 [− 26, 122], P = 0.204
Former smoker vs never smoker	− 10 [− 91, 71], p = 0.816	− 90 [− 249, 69], p = 0.268
Current smoker vs never smoker	− 35 [− 119, 50], p = 0.422	132 [− 34, 298], p = 0.120
Hypertension, yes vs no	− 89 [− 162, − 15], p = 0.180	− 103 [− 251, 45], p = 0.174
Antihypertensives, yes vs no	− 82 [− 183, 19], p = 0.110	− 104 [− 302, 95], p = 0.305
Diabetes, yes vs no	173 [11, 336], p = 0.037	361 [32, 691], p = 0.032
HDL-cholesterol, per mM	− 96 [− 177, − 15], p = 0.021	146 [− 20, 311], p = 0.085
LDL-cholesterol, per mM	− 33 [− 69, 2], p = 0.067	− 6 [− 79, 66], p = 0.869
Triglycerides, per mM	− 7 [− 31, 17], p = 0.556	− 55 [− 104, − 6], p = 0.027
Total Cholesterol per mM	− 44 [− 75, − 13], p = 0.005	− 13 [− 76, 51], p = 0.698
Antilipidemic, yes vs no	− 50 [− 156, 56], p = 0.357	265 [57, 473], p = 0.013
High− sensitivity CRP per mg/L	5 [− 1, 11], p = 0.077	31 [20, 42], p < 0.001
CD8 + cell count per 100 cells	15 [8, 23], p < 0.001	15 [0, 30], p = 0.044
CD4 + cell count per 100 cells	− 10 [− 22, 2], p = 0.094	− 13 [− 37, 10], p = 0.257
CD4 + nadir < 200cells/µL, yes vs no	14 [− 58, 85], p = 0.706	59 [− 82, 201], p = 0.412
HIV-RNA > 50copies/mL, yes vs no	385 [230, 539], p < 0.001	312 [3, 621], p = 0.048
Monocyte count per standard deviation	35.45 [1.65, 69.24] p < 0.001	104 [38, 170], p < 0.001

Association between independent variables and soluble CD163 in the left column and soluble CD14 in the right column

### Factors associated with soluble CD14 in PLWH

In unadjusted analyses in PLWH, age, diabetes, hsCRP, monocyte count, and CD8 + cell count, were associated with higher sCD14 concentrations. In adjusted analyses (Table 4), age, female sex*,* diabetes, hsCRP, use of antilipidemic, CD8 + cell count*,* and monocyte count were associated with higher sCD14 concentrations*.* Higher triglyceride concentration was associated with lower sCD14 concentrations*.* No association was found between smoking status, WHR, hypertension, HDL, LDL, cholesterol between CD4 + or low CD4 + nadir and sCD14 concentrations.

## Discussion

In a large study of PLWH and uninfected controls, PLWH had a higher prevalence of monocytopenia and lower mean monocyte counts than uninfected controls, and HIV status was independently associated with both lower monocyte count and with higher odds of monocytopenia after adjusting for confounders. In contrast, concentrations of monocyte activation markers were higher in PLWH than in uninfected controls, and HIV was independently associated with higher concentrations of monocyte activation markers.

Monocytopenia may be a result of decreased production from the hematopoietic stem cells in the bone marrow, increased destruction or due to altered distribution [30]. Monocytes and macrophages express CD4 receptors and CCR5 coreceptors and may become infected with HIV. In addition, myeloid precursor cells in the bone marrow also express these receptors and may, too, become infected with HIV [9, 11, 31–33]. Infection of myeloid cells with HIV is a cytotoxic event [34] which could interrupt the supply of new monocytes to the replenish the peripheral monocyte blood pool [35, 36]. Infection of monocytes and/or macrophages by HIV may also increase the turnover of mature cells, in a manner similar to CD4 T cells, and lead to lower numbers of circulating monocytes as they surge to replace end-stage tissue macrophages [37, 38]. In support of this, lower CD4 T cell counts as a marker of disease activity were associated with lower monocyte counts. However, CD4 T-cells counts were not associated with monocytopenia, and we did not find viral load to be associated with either monocyte counts or with monocytopenia. Of note, numbers were small as participants in the COCOMO study are mainly well-treated individuals, and fewer than five percent who had detectable viral replication, three out of five had viral loads under 200 copies/mL (data not shown). HIV binding to the monocyte CD4 receptor triggers monocyte activation and the migration of monocytes from the circulation into local tissues where they differentiate into resident macrophages and dendritic cells [9, 33]. Enhanced migration away from the blood stream may lower the number of circulating monocytes, especially under circumstances where monocyte production and macrophage life span are reduced.

Although the mean monocyte count was lower in PLWH than in the uninfected controls, the absolute difference was small (21/µL or ~ 5% of the mean monocyte count), and few had monocytopenia. Thus, any clinical implications of lower monocyte count in PLWH are likely negligible.

As reported by others [10, 11, 14, 16, 39–41], HIV was independently associated with higher concentrations of both sCD14 and sCD163, which in turn have been found to predict non-AIDS comorbidities as well as mortality in PLWH [10, 17–20, 23]. Activated monocytes are integral in the pathogenesis of vascular and pulmonary diseases, and soluble inflammation markers are thought to, in part, reflect inflammation in the vasculature as well as in the airways [14, 15, 42, 43]. Both vascular and pulmonary disease are strongly related to tobacco smoking, which is prevalent among PLWH [3, 44] where the risk of cardiovascular disease may be greater among PLWH who smoke than among uninfected smokers [7]. Increased immune activation has been speculated to be a potential mediator of this effect [7, 45, 46]. Although smoking status was strongly associated with monocyte counts in PLWH, we did not find evidence to support that smoking status is associated with soluble monocyte activation markers. This contrast with previous reports that have found higher levels of sCD14 and lower levels of sCD163 among smokers compared with nonsmokers [46, 47], and suggests that the detrimental health effects of tobacco smoking may not be mediated through monocyte activation. High concentrations of monocyte activation markers were also associated with higher mean monocyte counts. PLWH has lower monocyte counts than uninfected controls but higher concentrations of monocyte activation markers suggesting either higher production of monocyte activation markers per cell in PLWH or a contribution from macrophages located outside circulation[42, 48].

The main limitation to this study is the cross-sectional design, and we cannot conclude on causality. We used monocytes in peripheral blood from a drawn blood sample as representative for all monocytes and were not able to measure monocytes that may have adhered to the arterial vessel walls and have no information about tissue macrophages. Nor did we distinguish between different peripheral monocyte subsets. These limitations would, however, presumably have affected both PLWH and uninfected controls equally. Strengths of this study include the large, well-characterized study population, which was matched on sex and age, and the uniform collection and analysis of data which allowed us to explore the independent association of HIV serostatus with monocyte count.

## Concluding remarks

In conclusion, PLWH had lower mean monocyte counts and higher prevalence of monocytopenia, but higher concentrations of monocyte activation markers than uninfected controls. These associations remained after adjusting for confounders. Although monocytes concentrations were lower, the absolute difference was small, and any clinical implications of lower peripheral monocytes levels in PLWH are likely small. In contrast, high concentrations of monocyte activation markers have previously been implicated as drivers of non-AIDS comorbidity, and large-scale prospective studies aiming to determine any causal role of monocyte activation markers in the pathogenesis leading to non-AIDS comorbidity are warranted.

## Contribution to the field statement

Monocytes are drivers in the pathogenesis of inflammation-related diseases such as atherosclerosis. Monocytes express CD4 and CCR5 and can be infected by HIV. Increased levels of monocyte activation markers in persons living with HIV (PLWH) have previously been reported but the impact of HIV on circulating monocyte count, and the clinical significance of this in treated PLWH has not been explored. We investigated this in a large cohort of 871 PLWH from COCOMO and 4,355 matched uninfected controls. We also measured levels of soluble CD14 and CD163 that are systemic markers of monocyte activation.

Monocytopenia was more common in PLWH than in uninfected and this remained after controlling for confounding. Monocyte concentration was also lower in PLWH than in uninfected, but the absolute difference was small and likely without clinical relevance. Despite lower levels of circulating monocytes and more with monocytopenia, the level of soluble markers of monocyte activation was higher among PLWH.

We are the first to report that the monocyte count is associated with HIV status in well-treated PLWH. Our sample is large and well-characterized.

## Supplementary Information

**Additional file 1:** **Fig. S1.** Power plot. **Table S1. **Tables shows number individuals with missing information. **Table S2.** Association between risk factors and monocytosis.

## Data Availability

The datasets used and/or analysed during the current study are available from the corresponding author on reasonable request.

## Abbreviations

AIDS: Acquired Immunodeficiency Syndrome
BMI: Body mass index
cART: Combination anti-retroviral therapy
CGPS: Copenhagen General Population Study
COCOMO: Copenhagen Comorbidity in HIV infection
HDL: High-density lipoprotein
HIV: Human immunodeficiency virus
hsCRP: High sensitivity C-reactive protein
IQR: Interquartile range
LDL: Low-density lipoprotein
OR: Odds ratio
PLWH: People living with HIV
WHO: World Health Organization
WHR: Waist-hip ratio
