# Supplementary material for: Monocyte count and soluble markers of monocyte activation in people living with HIV and uninfected controls
Source: BMC Infect Dis. 2022 May 11;22:451. doi: 10.1186/s12879-022-07450-y (PMC9097376; doi:10.1186/s12879-022-07450-y)
Supplement: Supplementary file 1 — Additional file 1: Fig. S1. Power plot. Table S1. Tables shows number individuals with missing information. Table S2. Association between risk factors and monocytosis. [file 12879_2022_7450_MOESM1_ESM.docx]

**Additional file 1: FIGURE S1:** **Power plot**

***
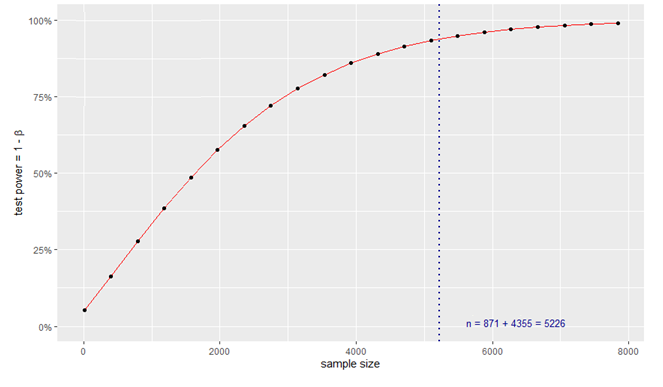
***

Power plot showing the relationship between sample size (X-axis) and Power (Y-axis) assuming a standard deviation of 135 and detectable difference of 18

Additional file 1: Table S1

Tables shows number individuals with missing information.

|  | **PLWH** | **Uninfected Controls** |
| --- | --- | --- |
| **Age in years*,* mean (SD)** | 0 | 0 |
| **Female sex, n (%)** | 0 | 0 |
| **Origin** | 13 | 57 |
| **BMI kg/m^2^, mean (SD)** | 4 | 11 |
| **WHR, mean (SD)** | 25 | 12 |
| **Smoking status** | 0 | 0 |
| **Hypertension, n (%)** | 53 | 97 |
| **Diabetes, n (%)** | 31 | 28 |
| **Antilipidemics, n (%)** | 0 | 129 |
| **Antihypertensives, n (%)** | 0 | 128 |
| **Undetectable, n (%)** | 0 | NA |
| **Low CD4 nadir, n (%)** | 0 | NA |

**Additional file 1:** **Table S2. Association between risk factors and monocytosis.**

|  | **Crude Odds Ratio** | **Adjusted Odds Ratio** |
| --- | --- | --- |
| **HIV, yes vs no** | 1.23 [0.68, 2.22], p=.488 | 0.98 [0.54, 1.80], p=.954 |
| **Age per decade** | 1.20 [0.97,1.48], p=.093 | 1.15 [0.92, 1.45], p=.224 |
| **Female sex, yes vs no** | 0.44 [0.18,1.09], p=.077 | 0.60 [0.23, 1.55], p=.290 |
| **Former smoker vs never smoker** | 1.21 [0.67,2.19], p=.533 | 1.06 [0.58, 1.96], p=.841 |
| **Current smoker vs never smoker** | 3.76 [2.14,6.59], p<.001 | 3.46 [1.96, 6.11], p<.001 |
| **Hypertension, yes vs no** | 1.76 [1.10,2.84], p=.020 | 1.45 [0.85, 2.46], p=.169 |
| **Antihypertensives, yes vs no** | 1.78 [1.01,3.13], p=.046 | 1.42 [0.76, 2.66], p=.276 |
| **Diabetes, yes vs no** | 0.97 [0.30,3.12], p=.965 | 0.63 [0.19, 2.08], p=.445 |
| **HDL-cholesterol, per mM** | 0.61 [0.35,1.06], p=.082 | 0.82 [0.46, 1.48], p=.515 |
| **LDL-cholesterol, per mM** | 0.94 [0.73,1.21], p=.634 | 0.92 [0.72, 1.17], p=.501 |
| **Triglycerides, per mM** | 1.14 [1.01,1.29], p=.037 | 1.08 [0.93, 1.24], p=.315 |
| **Total Cholesterol per mM** | 0.99 [0.80,1.22], p=.915 | 0.97 [0.78, 1.20], p=.752 |
| **Antilipidemic, yes vs no** | 1.12 [0.53,2.34], p=.772 | 0.79 [0.36, 1.72], p=.548 |
| **High-sensitivity CRP per mg/L** | 1.06 [1.04,1.08], p<.001 | 1.05 [1.03, 1.07], p<.001 |
| **CD8+ cell count per 100 cells** | 1.19 [1.10,1.30], p<.001 | 1.18 [1.08, 1.29], p<.001 |
| **CD4+ cell count per 100 cells** | 1.36 [1.18,1.57], p<.001 | 1.38 [1.19, 1.60], p<.001 |
| **CD4+ nadir < 200cells/µL, yes vs no** | 1.25 [0.42,3.75], p=.690 | 0.93 [0.29, 3.01], p=.909 |
| **HIV-RNA > 50copies/mL, yes vs no** | Model did not converge | Model did not converge |

*Associations between independent variables and monocytosis. Left column shows crude (unadjusted) odds ratios and right column shows adjusted odds ratios.*
